# Stem Cell Therapy: Past, Present, and Future Aspects

**DOI:** 10.3390/biomedicines14071443

**Published:** 2026-06-25

**Authors:** Ece Alim, Angelia Greenwell, Ryan Hess, Nicholas Blanco, Jorge H. Torres, Nurettin Sahiner

**Affiliations:** 1 Department of Biological Science, College of Arts & Sciences, Florida Gulf Coast University, Fort Myers, FL 33965, USA; ealim@fgcu.edu (E.A.); aigreenwell2016@eagle.fgcu.edu (A.G.); 2 Department of Bioengineering, Civil Engineering and Environmental Engineering, U.A. Whitaker College of Engineering, Florida Gulf Coast University, Fort Myers, FL 33965, USA; rghess2293@eagle.fgcu.edu (R.H.); nablanco1476@eagle.fgcu.edu (N.B.); jtorres@fgcu.edu (J.H.T.); 3 Department of Chemical Engineering, Faculty of Engineering, Canakkale Onsekiz Mart University, Terzioglu Campus, Canakkale 17100, Turkey

**Keywords:** stem cell therapy, tissue engineering, cell–tissue–organ, disease treatment, stem cell delivery

## Abstract

**Background/Objectives**: Stem cells with the ability to differentiate into other cell types and self-renewal afford a powerful apparatus for the healthcare system to replace and rejuvenate damaged tissues and organs in the treatment of various diseases. For the last few decades, stem cell therapy (SCT) has evolved from being an experimental approach to a recognized clinical treatment. SCT and regenerative medicine have garnered tremendous attention and become prominent tools, especially in treating chronic and acute disease and addressing organ failures, and in their repair and replacement, which are directly associated with human health, life, and longevity. **Methods**: In this review, after providing a brief history and need for the SCT, the employed delivery techniques utilizing various biomaterials, as well as recent developments in nanotechnological methods, are presented. It is focused on the current literature for the recent progress of stem cell therapy and tissue engineering for the application fields in neurological, ophthalmological, cardiovascular, orthopedic, and oncology, followed by the challenges associated with their applications. **Results**: In addition to safety concerns, challenges such as uncontrollable differentiations, genetic and epigenetic instability, limited cell survival and integration, immunological rejections, scaling and manufacturing drawbacks, as well as unpredictable behaviors and clinical limitations were reviewed. **Conclusions**: Future aspects with respect to regenerative medicine and tissue engineering, gene editing and personalized therapies, immunomodulation and anti-inflammatory applications, as well as neuroregeneration and treatment of neurodegenerative disorders are reflected.

## 1. Introduction

Stem cell therapy has emerged as one of the most promising strategies in regenerative medicine due to its potential to repair, replace, or regenerate damaged tissues and organs [1,2,3]. Over the past several decades, advances in stem cell biology, biomaterials, and translational medicine have significantly expanded the therapeutic landscape, enabling applications across a wide range of diseases, including neurological, cardiovascular, ophthalmological, orthopedic, and oncological conditions [4,5,6,7]. Despite remarkable progress, major biological, technical, and clinical challenges continue to limit the widespread and consistent success of stem cell-based therapies [8,9]. This review aims to provide a comprehensive overview of the past, present, and future aspects of stem cell therapy, with particular emphasis on emerging delivery strategies involving biomaterials and nanotechnology.

Stem cell therapy is a kind of renewing of cells and tissues or sometimes acknowledged as regenerative medicine where the stem cells or their different forms, e.g., the derivatives such as embryonic or pluripotent stem cells (PSC), or adult or tissue specific stem cells, and induced pluripotent stem cells (iPSC) can restore, reinstate or replace the impaired cells and tissues to regain the lost functions in the body [10,11,12]. Three main biological mechanisms for stem cell therapy are the replacement of the impaired cells, the delivery of the signals to foster the restoration, and the regulation of immune and inflammatory reactions [11,12,13]. As stem cell therapy focuses on the generation of cells and tissues rather than dealing with the symptoms of the ailment, it has many advantages in comparison to the common treatment methods, provided that the following concerns are eliminated. Firstly, no uncertainty and inefficiency for the target cells; secondly, no risks for immune reactions, infection, inflammation, and formation of different cell/tissue and tumor, etc., as well as other safety risks and complications are eliminated [10,11,12,13]. Finally, the economic and ethical considerations should be taken into consideration [14].

Stem cell-based therapies have shown significant potential in neurological disorders, where their therapeutic effects extend beyond cell replacement to include immunomodulation, neuroprotection, and enhancement of endogenous repair mechanisms. Applications in conditions such as Parkinson’s disease, spinal cord injury, stroke, and Alzheimer’s disease demonstrate the ability of stem cells to modulate the neural microenvironment, promote synaptic plasticity, and support functional recovery, although clinical translation remains limited by variability in outcomes and delivery challenges [15,16,17,18,19,20]. In ophthalmological diseases, stem cell therapies are being explored for vision restoration by regenerating corneal, retinal, and optic nerve tissues. Various stem cell types, including mesenchymal and pluripotent stem cells, have demonstrated the capacity to repair damaged ocular structures, reduce inflammation, and improve tissue homeostasis. Despite promising preclinical and early clinical outcomes, challenges such as immune response, integration efficiency, and long-term functionality remain key barriers [21,22,23]. Cardiovascular applications of stem cell therapy focus on repairing damaged myocardium and improving vascular function following injury. Stem cells, particularly mesenchymal stromal cells and induced pluripotent stem cell-derived cardiomyocytes, contribute to tissue repair primarily through paracrine-mediated mechanisms that promote angiogenesis, reduce apoptosis, and modulate inflammation. While clinical studies have generally demonstrated safety, with modest and often variable functional improvements, issues related to cell survival, engraftment, and long-term efficacy persist [24,25,26,27].

In orthopedic applications, stem cell therapies have shifted the paradigm from mechanical repair to biological regeneration of musculoskeletal tissues. Mesenchymal stromal cells play a central role through their ability to differentiate into bone and cartilage cells while also regulating the inflammatory microenvironment via their secretome. These therapies have shown promise in bone regeneration, cartilage repair, and osteoarthritis treatment, although optimization of delivery strategies and long-term outcomes remains necessary [28,29,30,31,32].

Stem cell-based approaches in oncology have evolved from supportive roles to advanced therapeutic strategies targeting tumor biology. In addition to hematopoietic stem cell transplantation, engineered stem cells are now used as delivery vehicles for targeted therapies, including oncolytic agents and immunotherapies. Emerging approaches such as cancer stem cell targeting and stem cell-derived drug delivery systems highlight the transformative potential of this field, although safety concerns such as tumorigenicity and immune complications remain critical challenges [33,34,35,36,37].

Collectively, these diverse applications highlight the broad and multifaceted therapeutic potential of stem cell-based approaches across multiple organ systems, while also underscoring the shared translational challenges that limit their clinical implementation.

## 2. Stem Cells Therapy Evolution and Challenges

### 2.1. Types of Stem Cells

Stem cells are the main or undifferentiated human cells with the ability to develop into different types of cells, such as muscle, bone, nerve, or brain, etc., with the capability of repairing impaired tissues. Stem cells function as the body’s main replacement and restoration system, dividing into other kinds of cells. Stem cells are mainly categorized by their origin and their capacity to differentiate. These include adult stem cells, which are multipotent or specific to certain tissues, embryonic stem cells (ESC) that are pluripotent, and induced pluripotent stem cells (iPSC). Stem cells can also be classified according to their potency, which is their ability to differentiate, ranging from totipotent to unipotent.

### 2.2. Emerging Endogenous Stem Cell Populations: Muse Cells and VSELs

In addition to commonly used stem cell types such as MSCs, ESCs, and iPSCs, some endogenous stem cell populations have recently attracted more attention because of their regenerative potential and possible advantages in clinical applications. Among these, multilineage-differentiating stress-enduring (Muse) cells and very small embryonic-like stem cells (VSELs) are becoming increasingly interesting because of their unique biological properties [38,39,40].

Muse cells are naturally occurring stem cells that can survive under stressful conditions and differentiate into different cell types while also showing immunomodulatory effects [38,39]. Compared with pluripotent stem cells, Muse cells appear to have a lower risk of tumor formation while still maintaining the ability to generate cells from different tissue types [38]. Studies suggest that Muse cells may support tissue repair not only by differentiating into damaged cells but also through paracrine effects and interactions with the surrounding tissue environment [39]. Their potential use has been investigated in several diseases, including spinal cord injury, ischemic stroke, myocardial infarction, and neurodegenerative disorders, with encouraging findings reported in both preclinical and early clinical studies [39,40].

VSELs are small populations of primitive stem cells that have been identified in adult tissues and may have pluripotent-like properties [41]. These cells have been suggested to play a role in tissue repair and regeneration because of their ability to self-renew and differentiate into different cell types [41]. However, some aspects of VSEL biology, including their exact identity and biological function, are still being discussed and require further investigation [41].

Although these cell populations are still being actively studied, their unique characteristics and lower tumorigenic potential may provide new opportunities for future regenerative medicine applications [38,39,40,41].

### 2.3. Therapeutic Mechanism of Stem Cells and Transition from Conventional Delivery to Advanced Biomaterials and Nanotechnology

The mechanism of stem cell therapy involves replacement of damaged cells via various mechanisms, e.g., activation of lineage-specific gene programs and signaling paths incorporating new cells into existing tissues, and release of active biomolecules such as growth factors, cytokines, chemokines, and exosomes, etc. Also, stem cell therapy needs to overcome excessive immune response and offset inflammation. Moreover, stem cell therapy is able to function and communicate appropriately with the microenvironments of the damaged tissue to improve vascularization, reduce oxidative stress, support internal repair and self-regulating processes, and adapt to the external changes.

Traditional stem cell delivery methods, most commonly direct injection or systemic infusion, have demonstrated limited efficacy due to low cell retention and lack of control over cell behavior at the target site [42,43]. As a result, there has been a paradigm shift toward the integration of biomaterials and nanotechnology to overcome these limitations [44,45]. Biomaterial-based delivery systems, such as hydrogels, cryogels, and injectable scaffolds, have been designed to mimic the native extracellular matrix, providing mechanical support and biochemical cues that promote cell survival, proliferation, and differentiation [46,47,48]. These platforms enable localized and sustained delivery of stem cells, as well as the co-delivery of growth factors or signaling molecules [49,50].

More recently, nanotechnology has emerged as a powerful tool to further enhance stem cell therapy. Nanomaterials can be engineered to protect cells during transplantation, modulate cell–material interactions, and enable controlled release of bioactive agents [51,52,53]. In addition, nano-enabled systems provide powerful tools for noninvasive molecular imaging and real-time tracking of transplanted cells, enabling monitoring of cell localization and fate in vivo. Beyond imaging, advances in nanomedicine have enabled targeted delivery strategies and the development of stimuli-responsive systems that react to specific features of the local microenvironment, such as pH, enzymatic activity, or redox conditions [54,55]. Together, these capabilities position nanotechnology as a key enabling platform for improving control, precision, and functional outcomes in stem cell-based regenerative therapies.

The convergence of stem cell biology with biomaterials science and nanotechnology represents a critical step toward the development of next-generation regenerative therapies. While early stem cell-based approaches focused primarily on cell transplantation, growing evidence indicates that therapeutic efficacy is strongly influenced by the engineered microenvironment, delivery strategies, and spatiotemporal control of biological cues. Integrating advances in biomaterial design and nanoscale engineering has therefore become essential for overcoming key translational barriers, including poor cell survival, limited functional integration, and inconsistent clinical outcomes. Understanding this interdisciplinary transition is therefore essential for advancing clinically effective and reproducible stem cell-based therapies [56,57].

### 2.4. Historical Development of Stem Cell Therapy

The concept of stem cells dates back to the early 20th century, when the term was first introduced to describe progenitor cells capable of self-renewal and differentiation [58]. Initial experimental and clinical evidence emerged from hematopoietic studies, culminating in the first successful bone marrow transplants in the 1950s and 1960s [59,60]. These early clinical applications laid the foundation for stem cell-based regenerative approaches and demonstrated the feasibility of cell-based therapies in humans.

The isolation of human embryonic stem cells (hESCs) in the late 1990s marked a major milestone, as these cells exhibited pluripotency and unlimited proliferative capacity [61]. Subsequently, the discovery of iPSCs revolutionized the field by enabling the reprogramming of somatic cells into pluripotent states without the ethical concerns associated with embryonic sources [62]. Mesenchymal stromal cells (MSCs) further accelerated translational efforts due to their immunomodulatory properties, relative ease of isolation, and favorable safety profile [63,64,65]. Historically, these cells have often been referred to as mesenchymal stem cells; however, according to the recommendations of the International Society for Cell & Gene Therapy (ISCT), the term “Mesenchymal Stromal Cells” is considered more scientifically appropriate because these cells may not consistently demonstrate true stem cell characteristics under all conditions [66]. Since the abbreviation MSC remains widely used in the literature, the term “Mesenchymal Stromal Cells (MSCs)” is used throughout this review for consistency with current recommendations [66].

Over time, the focus of stem cell therapy has shifted from proof-of-concept studies to disease-specific applications and clinical trials. While thousands of preclinical studies have demonstrated therapeutic potential, clinical outcomes have often been variable, highlighting the need for improved delivery strategies, enhanced cell survival, and better control of cell fate following transplantation [67,68,69].

### 2.5. Clinical Need, Challenges, and Limitations of Pluripotent Stem Cells

Despite major advances in modern medicine, many diseases, including neurodegenerative disorders, cardiovascular diseases, tissue injuries, and degenerative conditions, still lack effective regenerative treatments. Current therapeutic approaches often focus on symptom management rather than restoration of damaged tissues and organ function. Consequently, stem cell-based therapies have emerged as promising strategies because of their potential to support tissue regeneration and functional recovery [70,71]. Although pluripotent stem cells provide broad differentiation potential and offer important opportunities for regenerative medicine, several concerns still limit their clinical use. ESCs continue to raise ethical concerns because of their embryonic origin, while both ESCs and iPSCs have safety-related challenges that need further attention [72]. These concerns include genomic instability, risk of teratoma formation, uncontrolled differentiation, and abnormal cell growth [72,73]. In addition, differences in cell preparation methods and difficulties in obtaining consistent outcomes between studies may affect their translation into clinical applications [73]. A summary of the major limitations associated with pluripotent stem cell-based therapies is presented in Table 1.

## 3. Stem Cell Delivery Methods

### 3.1. Direct Injection Delivery Method

Direct injection remains one of the most widely used stem cell delivery strategies in regenerative medicine due to its technical simplicity, minimal invasiveness, and ability to achieve localized cell administration [11]. In this approach, stem cells—most commonly MSCs—are delivered directly into the target tissue or through locoregional routes such as intramyocardial, intramuscular, intra-articular, or trans-arterial injection, depending on the anatomical and pathological context as illustrated in [11,74]. Recent studies emphasize that locoregional delivery can improve tissue exposure and reduce off-target cell distribution compared to systemic infusion [74].

Following direct injection, transplanted stem cells do not uniformly engraft or differentiate; instead, they respond dynamically to local microenvironmental cues [74]. The extracellular matrix (ECM), soluble growth factors, cytokines, mechanical forces, and cell–cell interactions collectively regulate stem cell behavior by influencing gene expression patterns, paracrine signaling, and cytoskeletal organization [44,56]. Increasing evidence indicates that the therapeutic effects observed after direct stem cell injection are predominantly mediated by paracrine and immunomodulatory mechanisms rather than long-term cell engraftment or terminal differentiation [74,75].

Despite its advantages, direct injection is associated with several well-documented limitations. Rapid cell loss due to washout, mechanical stress, ischemia, and inflammatory responses at the injection site leads to low cell retention and short in vivo persistence [43,74]. Intravascular and intravenous injection routes further exacerbate these issues by causing nonspecific cell trapping in organs such as the lungs, liver, and spleen [43,74]. Advanced locoregional approaches, including image-guided trans-arterial delivery, have shown improved targeting efficiency but require specialized expertise and raise additional safety considerations [76]. Collectively, these challenges highlight the need for complementary strategies—such as biomaterial carriers and nano-enabled systems—to enhance cell retention, survival, and therapeutic efficacy following direct injection [47,56].

### 3.2. Use of Carriers: Hydrogel, Cryogels, and Polymer Matrices

**Figure 1:**
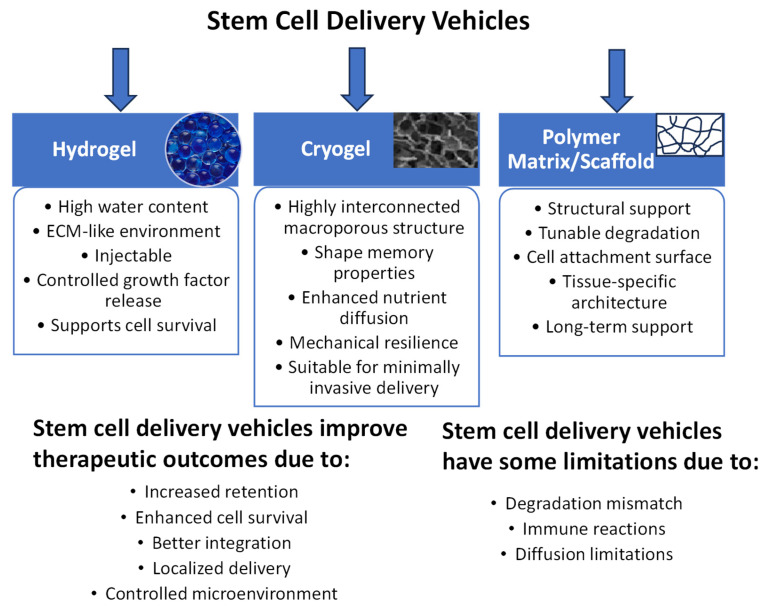
Representative biomaterial-based stem cell delivery vehicles, including hydrogels, cryogels, and polymer matrices. These systems provide structural support and regulate the local microenvironment to enhance cell retention, survival, and therapeutic performance. While hydrogels provide ECM-like properties and controlled release capabilities, cryogels improve nutrient diffusion and mechanical resilience, whereas polymer matrices offer long-term structural support and tissue-specific organization.

While direct injections enable localized delivery of stem cells, their clinical efficacy is frequently limited by poor cell retention, low survival rates, and hostile inflammatory microenvironments at the injection site [74,75]. To address these limitations, carrier-based delivery systems have been developed to provide structural support, protect transplanted cells from mechanical and immunological stress, and create a permissive microenvironment that enhances therapeutic performance [48,56]. Representative biomaterial-based delivery vehicles, including hydrogels, cryogels, and polymer matrices, and their functional properties are summarized in Figure 1.

Biomaterial carriers are designed to mimic key features of the native ECM, thereby regulating cell adhesion, survival, migration, and paracrine activity [44,56]. By embedding stem cells within a three-dimensional scaffold, carriers can reduce rapid cell washout and prolong cell residence time at the target site, leading to improved functional outcomes compared to bolus injection [77,78].

Among carrier systems, hydrogels are the most extensively investigated due to their high-water content, tunable mechanical properties, and excellent biocompatibility [46,48]. Hydrogels fabricated from natural polymers such as alginate, gelatin, and hyaluronic acid, as well as synthetic polymers including polyethylene glycol (PEG), allow encapsulation of stem cells under mild conditions that preserve cell viability and functionality [79,80]. Their porous network facilitates diffusion of oxygen, nutrients, and metabolites, supporting cell survival and sustained paracrine signaling following implantation [77].

In addition to serving as physical carriers, hydrogels can be engineered for controlled degradation and spatiotemporal release of therapeutic agents, including growth factors, cytokines, and extracellular vesicles, thereby enhancing regenerative signaling at the injury site [49,50]. Advanced designs, such as injectable, self-healing, and shape-memory hydrogels, cryogel, and gel and/or polymer matrices, further enable minimally invasive delivery and improved integration with host tissues [47,77]. Cryogels prepared under cryogenic conditions, e.g., below the freezing point of the solvent, are considered as special types of hydrogels with superior porosity and flexibility that can be used as stem cell delivery devices [56]. Additionally, polymeric matrices in any formulations, such as physical crosslinking, chemical crosslinking, or any modified forms as injectable systems, can also be used in the delivery of stem cells [46,58,80]. Any carrier systems, whether hydrogel, cryogels, or polymer matrices, have their own drawbacks due to the inherent nature of their sources, crosslinking method, and the agent used in crosslinking, and other problems such as biocompatibility, biodegradability, toxicity, and immune response to the degradation products, and so on. Despite these advantages, carrier-based systems are not without limitations. Excessive scaffold stiffness or thickness can impair nutrient diffusion and lead to hypoxia-induced cell death, while inadequate mechanical strength may result in premature deformation or collapse under in vivo loading conditions [48,81]. Moreover, improper degradation kinetics may either limit tissue integration or cause early loss of therapeutic benefit, and certain biomaterials may elicit local immune responses depending on their composition and implantation site [56,78]. These challenges underscore the importance of rational carrier design tailored to both the target tissue and the biological behavior of the delivered stem cells.

### 3.3. The Use of Nanotechnology and Nanomaterials in Stem Cell Therapy

Nanotechnology has emerged as a promising strategy to overcome key limitations in stem cell delivery, including low engraftment efficiency, poor spatial control, and the inability to monitor transplanted cells in vivo [82,83]. Current nano-enabled approaches can be broadly classified into three categories: (i) nanomaterials used as carriers or microenvironment modulators, (ii) nanotechnology-based tools for stem cell labeling and tracking, and (iii) active nano- and micro-scale devices, including nanorobots and nanoengineered platforms, designed to improve targeting precision and therapeutic control [82,83,84]. Nanomaterials can enhance stem cell delivery by improving cell retention, protecting cells from mechanical and inflammatory stress, and modulating cell–material interactions at the nanoscale [82]. A wide range of nanomaterials—including polymeric nanoparticles, lipid-based systems, mesoporous silica nanoparticles, gold nanoparticles, and magnetic nanoparticles—have been explored for their ability to interact with stem cells and deliver bioactive molecules in a controlled manner [82,85]. Importantly, nanoscale surface features and functionalized nanomaterials can influence stem cell adhesion, cytoskeletal organization, and mechanotransduction, thereby modulating paracrine activity and lineage commitment without necessitating terminal differentiation [84,86]. Rather than functioning as standalone delivery vehicles, nanomaterials are increasingly integrated into biomaterial scaffolds or hydrogels to create hybrid systems that combine structural support with nanoscale bioactivity [83,86]. Such nano-engineered microenvironments offer improved spatiotemporal control over biochemical and biophysical cues, which is critical for enhancing therapeutic consistency and functional outcomes in regenerative medicine applications [83].

Nanomaterials offer considerable potential to advance stem cell therapy by improving the regulation of cell fate, delivery efficiency, and post-transplantation monitoring. Nanoscale scaffolds and engineered surfaces, such as nano topographies, nanofibers, and nanocomposite hydrogels, can closely replicate key features of the native extracellular matrix. This biomimetic environment enhances stem cell adhesion, survival, and engraftment while providing physical and biochemical cues that guide differentiation toward specific lineages, including osteogenic, neural, cardiomyogenic phenotypes, and so on. Furthermore, nanocarriers further support therapeutic efficacy by enabling the targeted and controlled delivery of growth factors, genes, or small molecules to stem cells and their surrounding microenvironment, thereby strengthening paracrine signaling and allowing precise spatiotemporal modulation of biological cues. In addition, inorganic and metallic nanoparticles can facilitate noninvasive tracking of transplanted stem cells using MRI, optical, or nuclear imaging techniques, supporting real-time evaluation of biodistribution, persistence, and tissue integration in vivo [85,86,87]. Multifunctional theragnostic nano platforms may integrate these functions by combining therapeutic delivery and imaging within a single system, thereby enabling more adaptive and personalized treatment strategies. Despite these advantages, nanomaterials have important restrictions and safety concerns. Some of these drawbacks are toxicity, inducing oxidative stress, DNA damage, or membrane and mitochondrial injury that can compromise stem cell viability and function. Nanoparticle uptake may also disrupt normal differentiation and cause unintended or off-target lineage commitment, while persistent or poorly degradable materials can accumulate over time in organs such as the liver and spleen [54,83]. In addition, immune and inflammatory responses influenced by particle size, surface chemistry, and protein corona formation may reduce engraftment and raise systemic safety concerns, particularly after intravenous administration. From a clinical perspective, cell–nanomaterial products are difficult to characterize, standardize, and regulate; even minor variations in synthesis can alter biological responses, limit reproducibility, and slow translation.

#### 3.3.1. Nano-Enabled Labeling, Tracking, and Theragnostic Strategies

A major challenge in stem cell therapy is the inability to reliably monitor cell localization, survival, and fate after transplantation. Nano-enabled imaging agents and theragnostic nanoparticles provide noninvasive approaches for real-time tracking of transplanted stem cells, enabling correlation between biodistribution and therapeutic efficacy [54]. Nanoparticle-based stem cell labeling has been extensively investigated using magnetic, fluorescent, and multimodal contrast agents compatible with magnetic resonance imaging (MRI), positron emission tomography (PET), and optical imaging techniques [87].

Recent reviews emphasize that nano-enabled tracking is not merely a diagnostic tool but an important component of delivery optimization, allowing researchers to distinguish true engraftment from transient paracrine effects and to refine dosing and administration strategies [83,87]. Nevertheless, issues related to labeling stability, signal dilution during cell division, and long-term biosafety remain important considerations for clinical translation [87].

#### 3.3.2. Lipid Nanoparticles and Exosome-Mimetic Nanovesicles

Lipid nanoparticles (LNPs) and exosome-mimetic nanovesicles have recently gained more attention because of their potential use in stem cell-based therapies. LNPs are widely used as delivery systems because they are biocompatible, can carry nucleic acids and therapeutic molecules, and allow controlled delivery [88,89]. These systems may improve the stability and targeted delivery of genes, proteins, and signaling molecules while reducing degradation and unwanted effects in other tissues [88,89,90].

Exosome-mimetic nanovesicles have also become promising alternatives because they can mimic some properties of natural extracellular vesicles and may be easier to produce on a large scale [91,92]. These nanovesicles may support communication between cells, improve therapeutic cargo delivery, and help overcome some limitations associated with direct stem cell transplantation [91,92,93]. Although further studies are still needed, these approaches appear to be closer to clinical use than active nanorobotic systems [90,91,92,93].

#### 3.3.3. Nanorobots and Active Nano-/Micro-Devices

Beyond currently translatable nano-enabled systems, active micro- and nanorobotic systems represent an emerging future-oriented approach. These platforms are designed to navigate biological environments using external magnetic, acoustic, or chemical propulsion, potentially overcoming diffusion-limited transport and with the potential to improve targeting accuracy in complex tissues [94]. Biohybrid nanorobots, which integrate biological components with synthetic structures, have shown promise in preclinical models by combining enhanced motility with improved biocompatibility [95].

Despite their conceptual appeal, nanorobotic systems for stem cell delivery remain at an early developmental stage. Significant challenges, including precise control, scalability, long-term safety, and regulatory complexity, must be addressed before clinical application becomes feasible [94,95]. Accordingly, the current literature positions nanorobots as a future-oriented strategy rather than an immediately translatable delivery modality.

#### 3.3.4. Nanodevices and Nanoengineered Interfaces

Nanoengineered devices and surfaces provide an alternative strategy for influencing stem cell behavior by presenting instructive physical cues at the nanoscale. Nanotopographical interfaces have been shown to regulate stem cell adhesion, morphology, and differentiation through biophysical signaling mechanisms, supporting the development of next-generation delivery platforms that actively guide cell fate [84,86]. These nanodevices are most effective when combined with biomaterial-based delivery systems and evaluated using tissue-specific functional endpoints [86].

#### 3.3.5. Translational Considerations of Nano-Enabled Structures

The use of nano-enabled systems for stem cell therapy in medical care or clinical use requires the following considerations: (1) strict control of stem cell fate, (2) safety of nano-carriers in terms of manufacturability, monitoring, and disappearance, (3) reliability, and regulatory approval. Nano-enabled materials should be smarter, smart nanomaterials in terms of helping stem cells to survive, effectively reach the target site, ensure the development of the needed cell type, disappear safely after completing the task, provide no immune response, no harmful effects, and no accumulation within different parts of the body. The nanostructures to be used in nano-enabled stem cell therapy should also possess consistency and dependability in terms of precise size, reproducibility, scalability, and standardization for routine use. Across all nano-enabled delivery strategies, successful translation depends on reproducible results, predictable biodistribution and clearance, minimal immunogenicity, and clear regulatory pathways [55]. In nanomedicine, it is well accepted that increasing system complexity must be justified by measurable improvements in efficacy, safety, or monitoring capability [55]. Consequently, nano-enabled stem cell delivery approaches are most compelling as they directly address clearly defined capabilities and clinical limitations, including retention, inadequate targeting, or lack of post-transplantation monitoring [82,83].

### 3.4. Comparative Analysis of Stem Cell Delivery Strategies: Conventional vs. Biomaterial-Assisted vs. Nano-Enabled Systems

Different stem cell delivery approaches have distinct advantages and limitations regarding therapeutic effectiveness and clinical applicability. Direct injection remains the most commonly used strategy because it is technically simple, minimally invasive, and relatively easy to apply in clinical settings [79,80]. However, many studies have shown that directly injected cells often exhibit low retention rates and limited survival at the target site, reducing long-term therapeutic outcomes [43,80,81,82]. To overcome these limitations, biomaterial-assisted systems such as hydrogels, cryogels, and scaffold-based carriers have been developed [44,46]. These systems provide a supportive microenvironment for transplanted cells and can improve cell retention, survival, and localized therapeutic effects [48,49,50,83]. Compared with more complex delivery systems, biomaterial-based approaches currently appear more feasible for clinical translation because they can be produced more consistently and integrated more easily into existing therapeutic strategies [56,83,84,85,86,87].

On the other hand, ano-enabled systems provide additional advantages, including targeted delivery, controlled release of therapeutic agents, cell tracking, and theragnostic applications [54,82,83,84,85,86,87]. These features may improve the precision and effectiveness of stem cell therapies. However, despite their promising potential, several challenges remain, including large-scale manufacturing, long-term safety concerns, regulatory issues, and reproducibility of outcomes [55,95,96,97,98,99].

A comparison of the major characteristics, advantages, limitations, and translational status of these delivery approaches is summarized in Table 2. Overall, biomaterial-assisted approaches may currently represent a more practical near-term option for improving stem cell therapy outcomes, whereas nano-enabled systems have the potential to support the development of more advanced and personalized regenerative therapies in the future [55,56,95].

### 3.5. Challenges in Clinical Translation

Many stem cell studies have shown encouraging results in laboratory and animal studies; nonetheless, similar results are not always seen in clinical studies. One possible reason is that laboratory studies are usually performed under more controlled conditions, while patients are much more complex and can differ from one another in many ways. Factors such as age, disease stage, genetic background, and overall health may affect how patients respond to treatment and may lead to different outcomes between individuals [102,103]. Differences in stem cell preparation can also influence treatment results. Factors such as donor characteristics, cell source, culture conditions, manufacturing methods, and expansion procedures may affect cell behavior and therapeutic potential. In addition, treatment-related factors such as cell dose, delivery route, and timing of administration may also contribute to differences in clinical outcomes [56,80].

**Figure 2:**
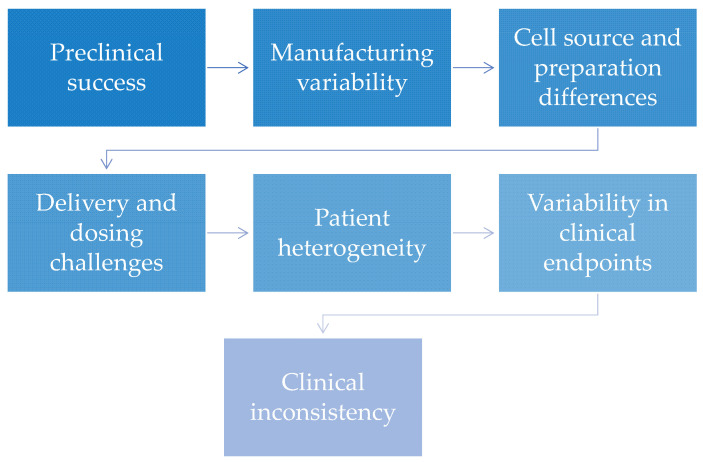
Factors contributing to the gap between preclinical success and clinical translation of stem cell therapies.

Another challenge is the difference between experimental models and real clinical settings. Preclinical studies often use more standardized conditions, while clinical studies involve more diverse patient populations and more complex disease conditions. Differences in study design and outcome measurements may also make results harder to compare across studies [104,105,106]. The major factors that may contribute to the gap between preclinical success and clinical outcomes are summarized in Figure 2. As can be seen, in addition to the variation in manufacturing, the source of the cells and their methods of preparation, as well as methods of delivery, patient heterogeneity, and so on, play a significant role in inconsistencies between preclinical success and clinical translation.

## 4. Stem Cell Therapy Applications

### 4.1. Neurological Diseases and Stem Cell-Based Therapies

**Figure 3:**
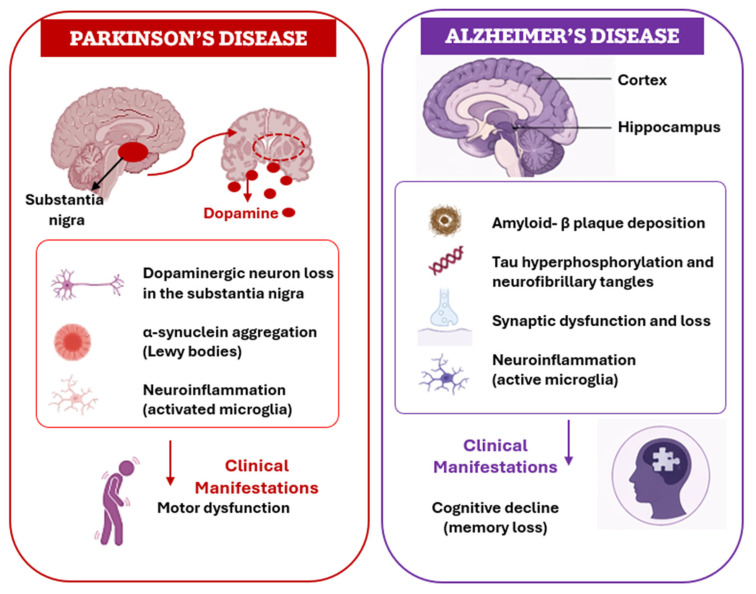
Parkinson’s and Alzheimer’s diseases, as neurodegenerative diseases, can be treated via stem cell therapy.

Parkinson’s and Alzheimer’s diseases are the most common and prevalent neurodegenerative diseases worldwide, as shown in Figure 3, and stem cell-based therapies can be very promising in their treatment by replacing lost neurons and slowing their progression.

#### 4.1.1. Stem Cell-Based Therapies in Parkinson’s Disease

Parkinson’s disease (PD) is characterized by the progressive loss of midbrain dopaminergic neurons projecting to the striatum, leading to dopamine deficiency and motor dysfunction, which makes dopaminergic cell replacement a rational therapeutic strategy [15,16]. The relatively selective degeneration of A9-type dopaminergic neurons and the well-established clinical responsiveness to dopamine further support the feasibility of restoring striatal dopaminergic input to improve motor outcomes [16,17].

Early transplantation studies using human fetal ventral mesencephalic (hfVM) tissue demonstrated that grafted dopaminergic neurons could survive long-term, innervate the host striatum, and provide sustained motor improvement in some patients [100,101].

However, variability in clinical outcomes due to differences in tissue preparation, graft composition, and immunosuppression protocols, along with complications such as graft-induced dyskinesia, highlighted the need for better standardization [96,97,100].

To overcome these limitations, the field transitioned toward pluripotent stem cell (PSC)-derived dopaminergic progenitors, including hESCs and iPSCs, enabling scalable and more consistent cell production [17,98]. Advances in differentiation protocols have improved lineage specificity and reduced risks associated with contaminating cell populations and tumorigenicity, while also supporting compliance with good manufacturing practice (GMP) and regulatory requirements [17,98,99].

Recent clinical developments in the year 2018–2025 range highlight the translational feasibility of PSC-derived dopaminergic progenitor therapies, including both iPSC- and hESC-based platforms [17,98,99]. Allogeneic iPSC-derived approaches have demonstrated evidence of graft survival, dopamine production, and acceptable safety profiles without tumor formation in early-phase clinical trials [107,108], while autologous iPSC strategies provide proof-of-concept for personalized therapies, albeit with significant challenges related to cost, scalability, and manufacturing complexity [17,109]. In parallel, hESC-derived dopaminergic progenitors have advanced as more standardized “off-the-shelf” products, showing encouraging early clinical outcomes, although long-term efficacy and consistency remain under investigation [99,110]. Recent studies also suggest that advanced delivery systems may improve the therapeutic outcomes of stem cell-based approaches in Parkinson’s disease [96,98,99,100,101]. Biomaterial-based systems such as hydrogels can provide structural support and create a more favorable microenvironment for transplanted dopaminergic cells [46,47,48,49,50,77]. In addition, nano-enabled delivery systems may help improve cell retention, protect transplanted cells from inflammatory conditions, and support more controlled release of neuroprotective molecules within the striatum [54,82,83,87]. These approaches may improve cell survival and may enhance integration of transplanted cells after administration [56,77,83].

#### 4.1.2. Stem Cell-Based Therapies in Spinal Cord Injury: Mechanisms, Clinical Progress, and Future Directions

##### Pathophysiological Rationale for Stem Cell Therapy in Spinal Cord Injury

Spinal cord injury (SCI) involves an initial mechanical failure followed by a secondary cascade including inflammation, oxidative stress, excitotoxicity, demyelination, glial scar formation, and neuronal loss, creating a highly inhibitory environment for axonal regeneration and functional recovery [18,111]. Due to this multifactorial pathology, stem cell-based therapies aim not only to replace lost neural cells but also to modulate inflammation, enhance trophic support, promote remyelination, and improve the regenerative microenvironment [18,112].

MSCs are among the most extensively studied cell types in clinical trials and may support recovery through mechanisms described above, including paracrine signaling and modulation of inflammatory responses [24,113]. Clinical studies report modest functional improvements in some patients; however, variability in cell preparation, dosing, and delivery methods limits definitive conclusions regarding efficacy [114].

Neural stem/progenitor cells (NS/PCs) offer greater potential for differentiation into neurons and glial cells, with preclinical studies demonstrating their ability to support synaptic integration and neural circuit remodeling [112,115]. Early phase clinical trials using human spinal cord-derived neural stem cells have demonstrated feasibility and acceptable safety, although efficacy outcomes remain preliminary [116].

Oligodendrocyte progenitor cells (OPCs), often derived from pluripotent stem cells, are designed to address demyelination by restoring myelin sheaths around surviving axons, thereby improving signal conduction [102]. Early clinical trials of OPC-based therapies demonstrated feasibility and acceptable safety profiles without evidence of tumor formation [102,117].

Current clinical studies are predominantly phase I/II trials focused on safety and feasibility, using standardized outcome measures such as the ASIA Impairment Scale [18,112]. Interpretation of clinical outcomes remains challenging due to heterogeneity in injury characteristics, timing of intervention, and treatment protocols [111,114].

##### Translational Challenges and Safety Considerations

Key translational challenges include limited cell survival and integration, particularly in chronic injury environments, as well as safety concerns such as tumorigenicity, immune responses, and neuropathic complications in pluripotent stem cell-derived therapies [102,111,112]. Although MSC therapies generally show favorable safety profiles, inconsistencies in manufacturing and characterization remain significant limitations [113,114].

Future strategies are expected to focus on combinatorial approaches integrating stem cell therapy with biomaterials, neuromodulation, and rehabilitation to enhance functional recovery [102,112]. Advances in iPSC technologies and improved GMP-standardized production, along with better patient stratification and robust clinical trial design, will be essential for achieving clinically meaningful outcomes [18,112,118].

#### 4.1.3. Stem Cell-Based Therapies in Stroke: Clinical Evolution, Mechanisms, and Future Perspectives

Stroke is a leading cause of long-term disability, and while acute reperfusion therapies benefit only a subset of patients, they do not restore lost neural tissue, which has driven the development of stem cell-based therapies focused on immunomodulation, neuroprotection, angiogenesis, and stimulation of endogenous repair rather than direct neuronal replacement [19]. Early clinical studies in the years 2011–2015 primarily investigated autologous bone marrow-derived cells, particularly mononuclear cells, administered intravenously in acute or subacute phases, demonstrating feasibility and safety but yielding inconsistent efficacy outcomes [103]. Subsequent randomized controlled trials confirmed safety but often failed to demonstrate consistent clinical benefit, highlighting the importance of factors such as cell type, dosing, timing, and endpoint selection [119,120].

More recent approaches have diversified into two main strategies: systemic administration of allogeneic cell products targeting inflammation in acute/subacute stroke, and intracerebral implantation techniques aimed at promoting local tissue repair in chronic stroke [119,121]. Systemic therapies, such as multipotent adult progenitor cells (MAPCs), have demonstrated safety in clinical trials, though consistent functional improvements remain difficult to establish without careful patient stratification [122,123].

In chronic stroke, intracerebral implantation strategies using modified MSCs, e.g., SB623 and neural stem cell lines, have shown encouraging safety profiles and potential functional improvements, although interpretation is limited by study design and sample size [124,125]. Advanced clinical trial designs incorporating blinding and sham controls have been increasingly adopted to reduce bias and improve reliability of outcomes [19,126,127,128]. Recent studies suggest that therapeutic effects may involve multiple mechanisms acting together, including paracrine signaling, immunomodulation, angiogenesis, and support of neural repair processes described above, where transplanted cells modulate immune responses, enhance angiogenesis, and promote synaptic plasticity and network remodeling, often through paracrine effects rather than long-term engraftment [19,129].

Meta-analyses have produced conflicting conclusions due to heterogeneity in study design, cell types, delivery routes, and patient populations, with some reporting no significant clinical benefit and others suggesting modest functional improvements alongside favorable safety profiles [130,131,132].

Future directions emphasize precision medicine approaches, including improved patient selection, mechanism-aligned outcome measures, standardized manufacturing processes, and combination therapies integrating rehabilitation, neuromodulation, or pharmacological interventions to enhance recovery [19,133].

#### 4.1.4. Stem Cell-Based Therapies in Alzheimer’s Disease: Mechanisms, Clinical Progress, and Future Directions

Alzheimer’s disease (AD) is characterized by synaptic dysfunction, neuroinflammation, amyloid-β accumulation, tau pathology, and progressive neuronal loss, and current therapies do not adequately address neuronal repair or neuroimmune dysregulation, motivating the development of stem cell-based approaches targeting immunomodulation, trophic support, and synaptic restoration [20,104,134,135]. As discussed above, common mechanisms such as paracrine signaling and immunomodulatory effects may play an important role in stem cell-based therapies for Alzheimer’s disease [20,105,135].

MSCs are the most clinically advanced platform due to their low immunogenicity and strong immunomodulatory properties, including the ability to regulate microglial and astrocytic activity, reduce pro-inflammatory signaling, enhance amyloid clearance, and support synaptic maintenance [20,106,136]. Neural stem/progenitor cells (NSs/NPs) have demonstrated potential in preclinical models by promoting synaptic plasticity, neurotrophic support, and modulation of amyloid- and tau-related pathways, often through indirect interactions with host neural cells [104,137,138,139]. iPSC-derived neural precursors have also shown promise in experimental models, with reported improvements in cognitive function and reductions in pathological markers, although translational challenges remain [140,141].

Preclinical studies consistently highlight several key mechanisms, including immunomodulation, synaptic rescue, and neurotrophic support, with cognitive improvements observed even in the absence of significant reductions in amyloid pathology [138,139,142].

Additionally, extracellular vesicles derived from MSCs have emerged as a promising cell-free therapeutic approach, demonstrating the ability to improve cognitive function and regulate neuroinflammation in animal models [143,144]. Clinical translation remains in early stages, with phase I trials demonstrating the feasibility and safety of intracranial and intracerebroventricular administration of MSCs in patients with mild-to-moderate AD [145,146,147]. More recently, randomized controlled trials of intravenous MSC therapies have confirmed safety and reported preliminary signals of clinical and imaging-based benefit, although larger studies are required for validation [148]. Despite these advances, significant challenges persist, including heterogeneity in cell sources and manufacturing protocols, limitations in delivery and biodistribution, variability in clinical endpoints, and incomplete understanding of therapeutic mechanisms [20,136,149,150]. Safety considerations, particularly regarding long-term monitoring and risks associated with pluripotent stem cell-derived products, remain critical for clinical translation [140,150].

**Figure 4:**
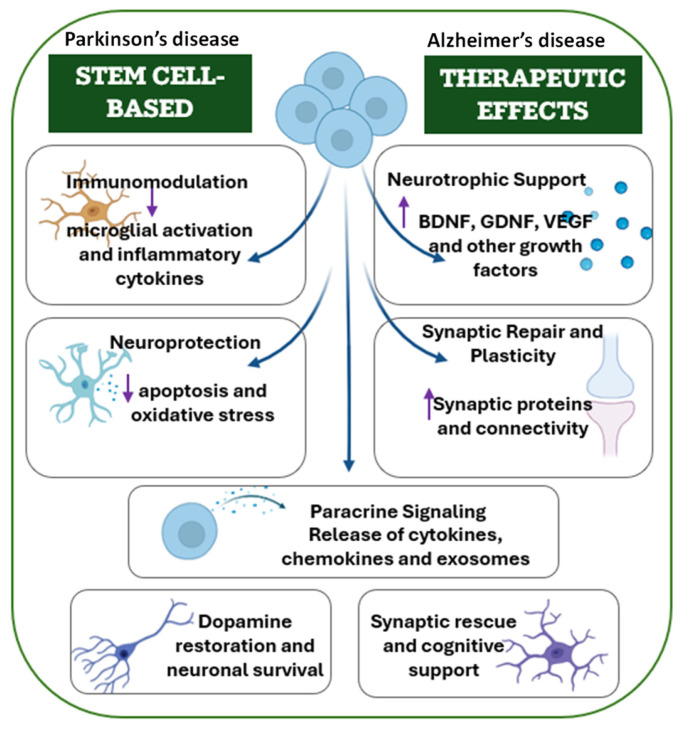
The use of stem cells in Parkinson’s and Alzheimer’s diseases.

Future directions emphasize standardized manufacturing, improved central nervous system delivery strategies, integration of biomarker-driven endpoints, and alignment of treatment approaches with disease stage and inflammatory profiles, with MSC-based therapies and extracellular vesicle strategies representing the most promising near-term clinical pathways, and Figure 4 summarizes the potential treatment the use of stem cells in Parkinson’s and Alzheimer’s diseases [20,107,108,109,110,143,148].

#### 4.1.5. Stem Cell-Based Approaches in Prion Diseases

Prion diseases are rare but severe neurodegenerative disorders caused by the accumulation of misfolded prion proteins, which result in neuronal damage, neuroinflammation, and progressive neurological decline. Currently available treatments are mainly supportive, and there are still no effective therapies that can stop disease progression. Stem cell-based approaches have recently attracted attention as possible treatment options because of their regenerative and immunomodulatory properties. Studies suggest that stem cells may support recovery through several mechanisms, including secretion of neurotrophic factors, regulation of inflammatory responses, and support of endogenous repair processes rather than direct replacement of damaged neurons. Mesenchymal stromal cells and neural stem cells have shown promising effects in experimental studies, including reduced neuronal damage and improvement of functional outcomes [151,152,153,154,155]. Although the results from experimental studies are encouraging, several limitations still remain. Limited understanding of disease mechanisms, poor long-term cell survival and integration, and lack of sufficient clinical evidence continue to restrict clinical application. Therefore, additional studies are needed to better evaluate the safety and therapeutic potential of stem cell-based approaches in prion diseases.

### 4.2. Ophthalmological Diseases and Stem Cell-Based Therapies

Stem cells are characterized by their capacity for self-renewal, proliferation, and differentiation into multiple cell types, making them highly suitable for regenerative therapies in ophthalmology [156]. In the eye, these properties are particularly relevant for restoring corneal integrity, repairing optic nerve damage, and treating retinal degenerative diseases.

#### 4.2.1. Stem Cells in Corneal Regeneration

The cornea plays a critical role in vision by acting as both a protective barrier and a refractive surface. Its transparency and function depend on the integrity of its three layers: epithelium, stroma, and endothelium [157,158]. The corneal epithelium is continuously renewed by limbal epithelial stem cells (LESCs), which reside in the limbus and generate transient amplifying cells that migrate toward the corneal surface [157,158,159,160]. Loss or dysfunction of LESCs leads to limbal stem cell deficiency (LSCD), a condition caused by injuries such as chemical burns or inflammatory diseases, resulting in impaired epithelial regeneration and vision loss [161,162,163,164]. While corneal transplantation remains the standard treatment, limitations such as donor shortage and immune rejection have driven the development of stem cell-based alternatives [157,162].

##### Mesenchymal Stromal Cells (MSCs) in Corneal Regeneration

**Figure 5:**
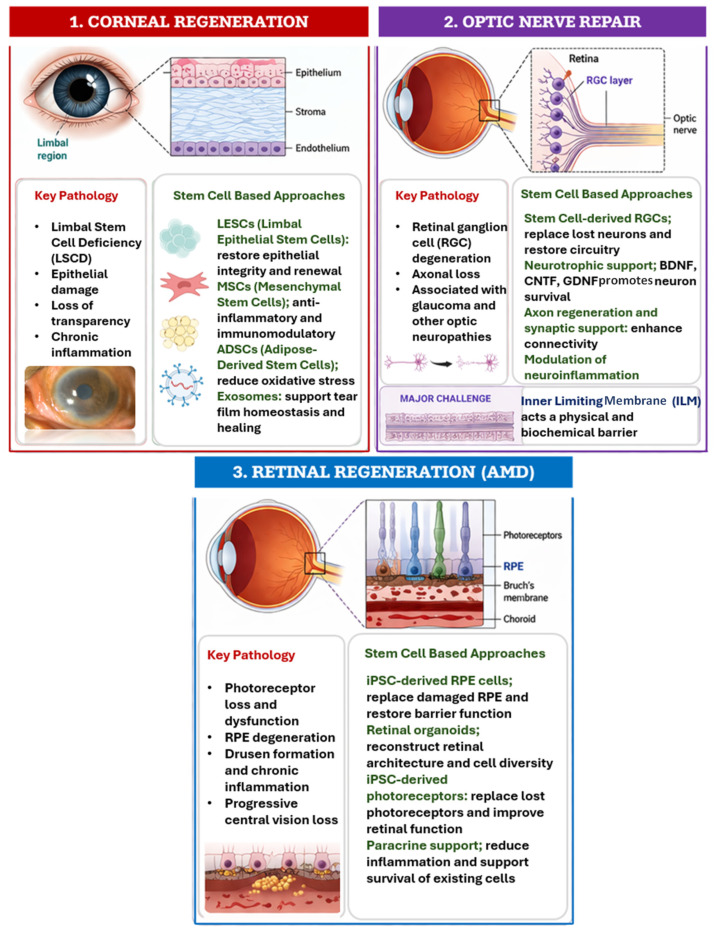
The use of stem cells in ophthalmologic applications.

MSCs, derived from sources such as bone marrow, adipose tissue, and umbilical cord, are widely studied due to their regenerative and immunomodulatory properties [165,166,167]. Bone marrow-derived MSCs (BM-MSCs) contribute to corneal repair by differentiating into stromal and epithelial-like cells and supporting wound-healing processes [168,169,170,171]. Clinical evidence demonstrates that subconjunctival BM-MSC administration can promote epithelial healing and improve clinical outcomes in severe ocular injuries, with minimal adverse effects [172]. Figure 5 shows the stem cell-based approaches in corneal regeneration.

Adipose-derived stem cells (ADSCs) are often considered advantageous compared to BM-MSCs due to their abundance, accessibility, higher yield, and minimally invasive harvesting procedures [173,174,175]. Experimental studies have demonstrated that ADSCs reduce oxidative stress, inflammation, and apoptosis in corneal injury models while promoting epithelial regeneration [176,177,178]. Moreover, modified ADSCs expressing growth factors such as insulin-like growth factor-1 (IGF-1) further enhance corneal repair by improving epithelial healing and reducing fibrosis and neovascularization [179]. In addition, MSC-derived exosomes represent a promising acellular therapeutic approach, contributing to improved tear secretion and reduced epithelial damage in dry eye disease models [180].

Other MSC sources, including umbilical cord-derived MSCs and dental pulp stem cells, have demonstrated the potential to contribute to corneal epithelial restoration and migrate to injury sites, indicating their regenerative capacity [181,182]. Additionally, conjunctiva-derived MSCs exhibit reduced immunogenicity and can differentiate into epithelial-like cells under optimized culture conditions [157,183].

##### Pluripotent Stem Cell Technology in Corneal Regeneration

Pluripotent stem cells, including iPSCs and ESCs, offer significant regenerative potential due to their ability to differentiate into all corneal cell types [62,184,185]. The iPSCs are generated by reprogramming somatic cells using transcription factors such as OCT4, SOX2, and c-MYC, enabling patient-specific therapies [62,186]. Differentiation protocols for iPSCs aim to mimic the limbal niche using signaling molecules such as BMP4, bFGF, and TGF-β inhibitors [187,188]. Advances in defined, xeno-free culture systems have improved reproducibility and clinical applicability [189,190,191]. iPSCs have been shown to generate corneal epithelial cells, endothelial cells, and stromal keratocytes, although differentiation efficiency varies depending on cell origin and epigenetic factors [192,193,194].

#### 4.2.2. Alternative Cell Sources in Corneal Regeneration

Alternative cell sources have also been explored to address limitations associated with conventional stem cell therapies. Skin keratinocyte stem cells (SKSCs), sharing a common ectodermal origin with corneal epithelial cells, have demonstrated the capacity for transdifferentiating into corneal epithelium. Tissue-engineered SKSC constructs have shown successful epithelialization and restoration of corneal transparency in LSCD models [195,196]. Oral mucosal epithelial cells (OMECs) represent another promising source due to their accessibility and high regenerative capacity. These cells exhibit multipotency and can differentiate into multiple lineages, including corneal epithelial-like cells, making them suitable for autologous transplantation strategies [197,198].

#### 4.2.3. Stem Cell-Based Optic Nerve Repair

Optic neuropathies are characterized by degeneration of retinal ganglion cells (RGCs), leading to irreversible vision loss due to the limited regenerative capacity of the central nervous system [199,200,201]. Stem cell-based approaches aim to restore visual function either through replacement of damaged RGCs or by promoting endogenous repair mechanisms, as illustrated in Figure 5. Transplantation of stem cell-derived RGCs has demonstrated the potential to integrate into host retinal tissue, establish synaptic connections, and partially restore visual function in experimental models [202]. In addition, MSC-derived exosomes have been shown to reduce apoptosis, enhance neurotrophic factor expression, and support RGC survival under stress conditions [203].

However, structural barriers such as the inner limiting membrane (ILM) significantly limit cell integration and functional recovery. Experimental studies indicate that disruption of this barrier can enhance donor cell engraftment and neurite extension, highlighting a key challenge for clinical translation [204,205]. Despite these limitations, stem cell-based strategies remain a promising approach for the treatment of glaucoma and other optic neuropathies.

#### 4.2.4. Stem Cell Therapy in Macular Degeneration

Age-related macular degeneration (AMD) is a leading cause of vision loss, particularly in individuals over 45 years of age [206,207]. The dry form (dAMD), which accounts for the majority of cases, is characterized by progressive degeneration of photoreceptors and retinal pigment epithelial (RPE) cells and currently lacks effective therapeutic options [208,209,210]. Stem cell-based therapies aim to replace damaged retinal cells and support retinal regeneration. Pluripotent stem cell-derived retinal organoids and RPE cells have demonstrated the capacity to integrate into host tissue, promote retinal reconstruction, and improve visual outcomes in preclinical models [211]. These approaches leverage the regenerative potential of stem cells to address limitations associated with conventional treatments [210,212]. Despite encouraging preclinical findings, challenges such as immune response, long-term integration, and functional stability remain to be addressed before widespread clinical application. Continued optimization of stem cell sources, differentiation protocols, and delivery strategies will be critical for successful translation into clinical practice, and use of SCT in AMD, along with key pathology, is summarized in Figure 5.

Overall, stem cell-based therapies offer significant promise in ophthalmology by enabling the regeneration of corneal, retinal, and optic nerve tissues. While both cellular and acellular strategies have shown encouraging results, key challenges related to immune compatibility, integration efficiency, and long-term functional outcomes remain. Addressing these limitations will be essential for the successful clinical translation of stem cell-based approaches in ocular diseases.

### 4.3. Cardiovascular Diseases and Stem Cell-Based Therapies

Cardiovascular and cerebrovascular diseases (CVDs) remain leading causes of mortality worldwide, primarily resulting from structural and functional abnormalities in the heart and vasculature [213,214,215]. Although current pharmacological and surgical interventions can alleviate symptoms, they are insufficient to restore damaged cardiac tissue, highlighting the need for regenerative therapeutic strategies [213].

MSCs have emerged as promising candidates due to their proliferative capacity, low immunogenicity, multilineage differentiation potential, and relatively favorable ethical profile [216,217]. However, MSC-based therapies are not without risks, including embolism, immune reactions, and potential tumorigenicity [218,219]. As discussed in the common mechanisms section above, therapeutic effects are believed to be mediated largely through paracrine signaling and immunomodulatory mechanisms rather than direct cell replacement. MSC-derived factors and extracellular vesicles may support tissue repair, angiogenesis, and reduction in inflammatory responses [220,221,222,223,224,225,226].

Clinical and preclinical studies have demonstrated that MSC-derived exosomes can localize to injured cardiac tissue, where they contribute to reduced apoptosis and inflammation, attenuation of adverse cardiac remodeling, and promotion of angiogenesis during tissue repair [27,227,228,229,230]. These findings support the growing view that acellular, exosome-based strategies may offer advantages in terms of safety and reproducibility. Numerous studies have investigated the therapeutic potential of both autologous and allogeneic MSCs in the treatment of acute myocardial infarction (MI). Clinical trials have demonstrated that MSC administration can improve cardiac function, including increases in ejection fraction (EF), reductions in arrhythmic events, and overall clinical stabilization in patients following MI [231,232]. Intracoronary delivery of autologous MSCs in patients with subacute MI has been associated with improvements in EF, reduced perfusion defects, and enhanced left ventricular remodeling [233]. Similarly, the Cardiosphere-Derived Autologous Stem Cells to Reverse Ventricular Dysfunction (CADUCEUS) trial demonstrated a significant reduction in myocardial scar size and increased viable cardiac tissue, although no consistent improvements in EF or ventricular volumes were observed [234]. Preclinical studies in both small and large animal models further support the therapeutic potential of MSCs in ischemic heart disease, demonstrating improved cardiac function, reduced infarct size, decreased myocardial apoptosis, and enhanced angiogenesis [235,236,237,238]. Different delivery routes, including intramyocardial, intracoronary, and intravenous administration, have been shown to contribute to reverse remodeling, improved cardiac performance, and mitigation of ischemia–reperfusion injury [239,240,241]. In parallel, pluripotent stem cell-based approaches have gained increasing attention. Transplantation of iPSC-derived myocardial patches has demonstrated significant improvements in cardiac function and increased vascular density in porcine infarction models, with no reported evidence of tumor formation or fatal arrhythmias during the study period [242]. Similarly, iPSC-derived cardiac spheroids have been shown to improve EF and reduce infarct size in large animal models, although arrhythmogenic effects such as tachycardia have been reported [243].

Meta-analyses of preclinical studies indicate that iPSC-derived cardiomyocyte therapies significantly improve left ventricular EF and fractional shortening, although no consistent effects on mortality or ventricular arrhythmias have been observed [244]. In addition, iPSC-derived endothelial cells and their exosomes have shown therapeutic potential by reducing cardiomyocyte apoptosis, improving cardiac function, and promoting angiogenesis [245]. Combined transplantation of iPSC-derived endothelial cells and cardiomyocytes has further enhanced cardiac repair, resulting in improved functional outcomes and increased vascular formation in experimental models [246].

### 4.4. Orthopedic Applications and Stem Cell-Based Therapies

Although cardiovascular applications have highlighted the systemic regenerative potential of stem cell-based therapies, their role in musculoskeletal repair has further expanded the field by addressing localized structural and functional tissue damage. Orthopedic surgery has traditionally focused on mechanical restoration using fixation devices, prosthetics, and grafts. While these approaches improve structural stability, they fail to tackle the underlying biological mechanisms of tissue degeneration and impaired healing. This limitation has driven a paradigm shift toward regenerative strategies, particularly stem cell-based therapies aimed at restoring functional musculoskeletal tissue [32]. MSCs, derived from sources such as bone marrow, adipose tissue, and umbilical cord, play a central role in this transition due to their capacity for multilineage differentiation, immunomodulation, and homing to injury sites [28,29,30]. Importantly, MSCs are now recognized not merely as structural building blocks but as dynamic biological regulators that orchestrate tissue repair through the secretion of bioactive molecules collectively known as the secretome [247].

#### 4.4.1. Bone Regeneration

The treatment of critical-sized bone defects remains a major clinical challenge, as these injuries exceed the body’s intrinsic regenerative capacity. Contemporary regenerative strategies are guided by the “Diamond Concept,” which integrates osteogenic cells, growth factors, scaffolds, and mechanical stability to facilitate bone healing [248]. MSCs contribute to this process through differentiation into osteoblasts responsible for bone matrix formation, while also modulating the local microenvironment [28]. Their activity is regulated by inflammatory signaling, which influences migration, differentiation, and regenerative potential. In addition to direct differentiation, MSCs promote angiogenesis via their secretome, supporting vascularization and tissue maturation [30]. A key feature of MSCs is their homing capacity, enabling migration to sites of injury and inflammation. Once localized, MSCs promote a shift from a pro-inflammatory to a pro-regenerative environment, enhancing tissue repair and reducing the risk of non-union [249].

#### 4.4.2. Cartilage Repair and Osteoarthritis

Articular cartilage repair has historically been limited by its avascular nature and poor intrinsic healing capacity. Early approaches, such as microfracture, aimed to recruit endogenous progenitor cells but often resulted in fibrocartilage formation rather than functional hyaline cartilage [250]. Similar to other stem cell applications, therapeutic effects in orthopedic conditions may involve common mechanisms described above, including paracrine signaling, regulation of inflammatory responses, and support of tissue repair. MSCs migrate to sites of joint injury and modulate the inflammatory environment, suppressing catabolic pathways associated with cartilage degradation [247]. The therapeutic effects are largely mediated by the MSC secretome, which includes growth factors and cytokines that promote tissue regeneration and support integration of cartilage with underlying bone structures [31].

Osteoarthritis (OA) is now recognized as a complex inflammatory disease affecting the entire joint rather than a purely degenerative condition. This paradigm shift has significantly influenced stem cell-based therapeutic strategies. Early MSC-based approaches focused on direct differentiation into chondrocytes to replace damaged cartilage [251]. However, limited long-term engraftment led to the “hit-and-run” hypothesis, suggesting that therapeutic benefits primarily arise from transient paracrine signaling rather than sustained cell integration [252]. Current approaches emphasize the role of MSCs as “biological factories,” secreting anti-inflammatory and regenerative factors that shift the joint environment from a catabolic to an anabolic state [253]. These effects include suppression of inflammatory cytokines, inhibition of matrix degradation, and promotion of tissue repair. Clinical studies and recent meta-analyses have demonstrated that MSC-based therapies can provide pain relief and functional improvement in OA patients, although outcomes remain variable [254,255].

Emerging strategies focus on cell-free approaches, particularly exosome-based therapies, which deliver targeted molecular signals while minimizing risks associated with live cell transplantation [256]. In addition, gene-editing technologies such as Clustered Regularly Interspaced Short Palindromic Repeats (CRISPR) are being explored to enhance the regenerative and anti-inflammatory potential of MSCs, enabling more precise therapeutic interventions [257].

### 4.5. Cancer Therapy and Stem Cell-Based Approaches

Stem cell applications in oncology have evolved from supportive roles in hematopoietic recovery to advanced therapeutic strategies targeting tumor biology. Historically, hematopoietic stem cell transplantation (HSCT) was primarily used to restore bone marrow function following high-dose chemotherapy [33]. The introduction of peripheral blood stem cells (PBSCs), mobilized by factors such as granulocyte colony-stimulating factor (G-CSF), significantly improved clinical outcomes by accelerating engraftment and reducing recovery time [34,35]. Beyond hematopoietic reconstitution, stem cells are increasingly being explored as targeted therapeutic delivery vehicles. MSCs, for instance, can be engineered to exploit their intrinsic tumor-homing capacity, enabling site-specific delivery of oncolytic viruses or prodrug-activating enzymes within the tumor microenvironment [36]. In parallel, chimeric antigen receptor (CAR)-T cell therapy has emerged as a major advancement, utilizing engineered immune cells to selectively recognize and eliminate malignant cells [258]. Although paracrine signaling is frequently described as one of the major mechanisms responsible for stem cell therapeutic effects, these effects are mediated by different bioactive molecules, including growth factors, cytokines, extracellular vesicles, and regulatory microRNAs. Representative molecules involved in different disease systems are summarized in Table 3.

Emerging strategies focus on iPSCs as scalable platforms for generating “off-the-shelf” immune cells, enabling standardized and rapid therapeutic applications [259]. In addition, genetic engineering of hematopoietic stem cells offers the potential for long-term immune surveillance and sustains anti-tumor activity, reducing the risk of relapse [260].

The primary mechanisms underlying stem cell therapeutic effects across different systems are compared in Table 4.

#### 4.5.1. Cancer Stem Cells

The cancer stem cell (CSC) hypothesis has significantly reshaped the understanding of tumor biology, proposing that tumor initiation, progression, and recurrence are driven by a subpopulation of cells with self-renewal and differentiation capacity [37]. Conventional therapies such as chemotherapy and radiotherapy primarily target rapidly dividing cells but often fail to eliminate quiescent CSCs, contributing to therapeutic resistance and tumor relapse [261]. Current research focuses on identifying CSC-specific markers, including CD44 and CD133, across various malignancies [262]. Therapeutic strategies aim to disrupt key signaling pathways that regulate CSC maintenance, such as Notch, Wnt, and Hedgehog pathways [263]. MSCs are increasingly explored as targeted delivery systems for CSC-directed therapies, leveraging their tumor-homing properties to transport therapeutic agents directly into the tumor microenvironment [36]. Future approaches integrate iPSC-derived immune cells and CRISPR-based gene editing technologies to selectively eliminate CSC populations, offering the potential for more durable therapeutic outcomes [259].

#### 4.5.2. Stem Cell-Based Drug Delivery

Stem cell-based drug delivery systems have evolved from passive carriers to engineered, site-specific therapeutic platforms. Early approaches utilized stem cells as “Trojan horses,” loading them with chemotherapeutic agents such as paclitaxel or doxorubicin to enhance tumor targeting while reducing systemic toxicity [264]. However, these methods were limited by premature drug release and reduced cell viability. Recent developments focus on genetically engineered stem cells capable of producing therapeutic agents directly at tumor sites. Neural stem cells (NSCs), for example, have been used in clinical studies to deliver prodrug-activating enzymes to glioblastoma, enabling localized conversion of non-toxic compounds into cytotoxic agents within the tumor microenvironment [265]. Similarly, hematopoietic stem cells (HSCs) are being engineered for sustained therapeutic protein delivery, demonstrating potential in systemic and metabolic disease contexts [266].

Future directions emphasize cell-free delivery systems, particularly engineered exosomes, which can be loaded with therapeutic molecules such as microRNAs or CRISPR/Cas9 components. These systems combine the targeting precision of stem cells with improved safety and stability, reducing risks associated with live-cell transplantation [267]. In addition, iPSC-derived platforms are being developed as scalable and standardized delivery systems for clinical applications [268].

#### 4.5.3. Safety Concerns

Despite their therapeutic potential, stem cell-based oncology approaches present significant safety challenges. One of the primary concerns is tumorigenicity, particularly associated with pluripotent stem cells such as ESCs and iPSCs, which possess high proliferative capacity and may form teratomas if not fully differentiated [269]. In addition, prolonged in vitro expansion and manipulation of MSCs may lead to genetic instability, increasing the risk of malignant transformation [270]. Clinical observations have reported rare cases of abnormal tissue formation following stem cell transplantation, underscoring the need for long-term monitoring [271]. Another critical issue is the dual role of MSCs within the tumor microenvironment. While engineered MSCs may exert anti-tumor effects, unmodified MSCs can contribute to tumor progression by differentiating into tumor-associated fibroblasts and promoting angiogenesis, thereby supporting tumor growth and metastasis [272,273].

Immunological risks also remain significant, particularly in allogeneic transplantation settings. HSCT and PBSC therapies are associated with complications such as graft-versus-host disease (GvHD), as well as long-term adverse outcomes including secondary malignancies, cardiovascular complications, and endocrine dysfunction [274,275,276]. To mitigate these risks, modern clinical protocols incorporate advanced strategies such as selective T-cell depletion and CD34+ cell enrichment to improve graft safety [277]. Future developments include engineered “kill-switch” systems that allow controlled elimination of transplanted cells, as well as the increasing adoption of cell-free therapeutic approaches to minimize tumorigenic risk. Integration of gene-editing technologies and real-time monitoring systems is expected to further enhance the safety and precision of stem cell-based cancer therapies.

Stem cell-based approaches in oncology have progressed from supportive therapies to highly targeted and engineered treatment strategies. Advances in CSC targeting, drug delivery systems, and immunotherapy highlight their transformative potential. However, challenges related to safety, tumorigenicity, and immune responses remain critical barriers. Continued innovation in gene editing, exosome-based therapies, and standardized cell platforms will be essential to fully realize the clinical potential of stem cell-based cancer treatments. A comprehensive summary of stem cell applications across different disease systems is provided in Table 5.

### 4.6. Clinical Applications Across Different Disorders

Stem cell therapies are currently being investigated for many different diseases because of their regenerative potential and their ability to influence tissue repair and immune responses. Depending on the disease type, stem cells may support treatment through different mechanisms such as cell replacement, paracrine signaling, angiogenesis, and regulation of inflammatory pathways.

In neurological disorders, stem cells have been investigated to support neuronal repair and improve functional recovery. In ophthalmological applications, they have been used for the regeneration of corneal and retinal tissues. Cardiovascular studies mainly focus on improving cardiac repair and vascular function after injury. In orthopedic diseases, stem cell-based therapies have shown potential in bone and cartilage regeneration, while in cancer-related applications, stem cells have been explored for targeted drug delivery and immune-related therapies [10,16,17,18,19,20,21,22,23,24,25,26,27,28,29,30,31,32,33,34,35,36,37]. Stem cell therapy spans a wide spectrum of preclinical and clinical applications, from established hematopoietic stem cell transplantation to experimental regenerative approaches using mesenchymal, neural, and pluripotent stem cells. Preclinically, embryonic stem cells and induced pluripotent stem cells are being explored for replacement of lost cells and tissue repair in models of central nervous system injury (stroke, spinal cord injury, Parkinson’s disease) [84,85,86,87,88,89,90,91,92,93,98,99,100,101,107,108,109,110], myocardial infarction, liver and pancreatic disease, and retinal degeneration, where they can generate new neurons, cardiomyocytes, hepatocytes, beta cells, or retinal cells and integrate into host circuits [215,216,217,218]. Mesenchymal stem cells from bone marrow, adipose, and other postnatal tissues are being tested extensively in animal models of musculoskeletal disorders, autoimmune and inflammatory diseases, cardiovascular and cerebrovascular injury, and neurodegeneration, acting mainly via paracrine immunomodulatory and trophic effects rather than durable engraftment. Clinically, hematopoietic stem/progenitor cell transplantation is standard of care for many hematologic malignancies and inherited blood disorders, while mesenchymal stem cell products have progressed into trials and limited approvals for conditions such as graft-versus-host disease, Crohn’s disease, certain orthopedic indications, and exploratory studies in stroke, myocardial infarction, and neurodegenerative diseases [84,85,86,87,88,89,90,91,92,93,119,120,121,122,123,124,125,126,127,128,129,130,131,132,133,239,240]. Early phase trials with pluripotent stem cell-derived products—such as dopaminergic neurons for Parkinson’s disease, oligodendrocyte progenitors for spinal cord injury, and retinal pigment epithelium for macular disease—demonstrate feasibility but highlight challenges related to genomic stability, tumorigenicity, immune compatibility, and manufacturing consistency, so that much of the field is still in translation from robust preclinical proof-of-concept to broadly effective and safe clinical therapies.

Although many studies have reported promising findings, several limitations remain, including differences in treatment outcomes, limited long-term data, and challenges associated with clinical translation. Continued improvements in delivery methods and treatment strategies may help increase the effectiveness of stem cell therapies in future clinical applications.

## 5. Challenges in Stem Cell Therapies

Despite the significant therapeutic potential of stem cell-based approaches, several critical challenges limit their safe and effective clinical translation. These challenges include uncontrolled differentiation, tumorigenicity, genetic instability, poor cell survival and integration, immunological rejection, and difficulties in large-scale production.

### 5.1. Uncontrolled Stem Cell Differentiation

One of the most fundamental limitations of stem cell therapies is the risk of uncontrolled differentiation following transplantation. PSCs, such as ESCs and iPSCs, possess the ability to differentiate into any cell type; however, this same property predisposes them to form teratomas when differentiation is not tightly controlled [278,279]. Even small populations of residual undifferentiated cells can lead to abnormal tissue formation, representing a major safety concern in clinical applications. While MSCs are generally considered safer, they may exhibit aberrant differentiation under certain conditions [11].

Controlling differentiation in vivo remains challenging due to the complex interactions between transplanted cells and the host microenvironment, including extracellular matrix signals and immune responses [280]. Strategies such as inducible gene expression systems and biomaterial-based scaffolds have been explored to improve control over stem cell fate, although these approaches require further refinement [281,282].

### 5.2. Genetic and Epigenetic Instability

Long-term in vitro expansion of stem cells introduces significant risks of genetic and epigenetic instability. Prolonged culture conditions expose cells to oxidative stress, mechanical strain, and replication-associated errors, leading to DNA damage and chromosomal abnormalities [278,279]. Common genetic alterations include aneuploidies, trisomies, and structural rearrangements, which may confer proliferative advantages but compromise safety and differentiation capacity [283,284]. These abnormalities can disrupt key regulatory pathways and increase susceptibility to malignant transformation [285,286].

In addition to genetic mutations, epigenetic changes—such as altered DNA methylation and histone modifications—can further destabilize stem cell behavior and impair controlled differentiation [287,288]. These changes may persist after transplantation, contributing to unpredictable outcomes and increased tumorigenic risk. To mitigate these risks, advanced monitoring techniques, including next-generation sequencing and cytogenetic screening, are essential for detecting genomic alterations prior to clinical use [289,290]. Maintaining genomic integrity remains a critical requirement for the safe application of stem cell therapies.

### 5.3. Limited Cell Survival and Integration

A major barrier to therapeutic success is the poor survival and integration of transplanted stem cells. Studies indicate that a substantial proportion of transplanted cells fail to survive shortly after administration due to immune responses, inflammation, and unfavorable microenvironments [291]. Additionally, inadequate vascularization, insufficient extracellular matrix support, and lack of appropriate signaling cues limit engraftment and functional integration into host tissues [292,293]. These limitations significantly reduce the regenerative potential of stem cell therapies.

Strategies to improve survival and integration include preconditioning of stem cells, use of biomaterial scaffolds, and enhancement of local microenvironments to support cell adhesion and function [294,295]. Immunomodulatory approaches are also being explored to reduce rejection and improve long-term engraftment [296,297].

### 5.4. Immunological Rejection

Immunological rejection remains a major concern and may occur even in autologous stem cell settings due to alterations in antigen expression. Reprogramming and differentiation processes can alter antigen expression, leading to immune recognition and rejection of transplanted cells [298]. Allogeneic stem cell therapies carry additional risks, including graft-versus-host disease (GvHD) and long-term complications such as secondary malignancies and organ dysfunction [274,275,276].

To address these challenges, strategies such as improved cell purification, immune tolerance induction, and transient immunosuppression are being developed. Advances in gene editing and cell engineering may further reduce immunogenicity and enhance compatibility between donor and recipient cells [299].

### 5.5. Scaling and Manufacturing Challenges

The large-scale production of clinically relevant stem cells presents significant technical and biological challenges. Expansion of stem cells while maintaining pluripotency and genomic stability is difficult, as prolonged culture may lead to variability in cell quality and function [300].

Advanced culture systems, such as bioreactors and suspension-based platforms, have been developed to improve scalability; however, these systems require precise control of environmental conditions to prevent differentiation and genetic drift [301,302]. Standardization of production protocols and quality control measures is essential to ensure reproducibility and safety in clinical applications.

### 5.6. Unpredictable In Vivo Behavior

Stem cell behavior in vivo often differs significantly from in vitro expectations due to the complexity of the host environment. Factors such as cytokine signaling, immune activity, and extracellular matrix composition can alter cell fate, survival, and therapeutic outcomes [303,304].

To better predict these responses, advanced preclinical models, including organ-on-chip systems, are being developed to simulate physiological conditions more accurately and improve translational success [305].

### 5.7. Tissue-Specific and Clinical Limitations

Stem cell therapies also face tissue-specific challenges that limit their effectiveness across different applications. For example, successful regeneration depends heavily on the integrity of the stem cell niche, which provides essential biochemical and mechanical signals for maintaining stem cell function [306,307,308].

In ocular applications, disruption of the limbal niche impairs stem cell maintenance and regeneration, highlighting the importance of microenvironmental support [309]. Similarly, iPSC-based therapies continue to face challenges such as low differentiation efficiency, tumorigenic risk, and high production costs, which limit widespread clinical use [306,310,311,312,313,314,315].

In neural and retinal applications, poor survival and integration of transplanted cells, particularly retinal ganglion cells, remain major obstacles due to the complexity of neural circuitry [201,316,317]. Clinical gaps are also evident in diseases such as age-related macular degeneration and ischemic heart disease, where stem cell therapies have shown promise but require further validation to achieve consistent and reliable outcomes [318,319].

### 5.8. Aging, Cellular Senescence, and Programmed Cell Death

Aging has become an important factor in stem cell research because it may affect how stem cells function and respond after transplantation. As stem cells become older, their ability to proliferate, self-renew, and differentiate may gradually decrease, which can reduce their regenerative potential and therapeutic effectiveness [306,307].

Cellular senescence is another important issue because senescent cells remain alive but lose their ability to divide normally. These cells may also release inflammatory molecules and other signaling factors that can negatively affect nearby tissues and alter the surrounding microenvironment [308,309].

Programmed cell death pathways, including apoptosis and other regulated cell death mechanisms, are also involved in stem cell survival and tissue homeostasis. Excessive cell death after transplantation may reduce cell survival and may negatively affect long-term therapeutic outcomes [310]. A better understanding of aging-related changes and cell survival mechanisms may help improve stem cell function and may support the development of more effective regenerative therapies in future applications.

## 6. Clinical Needs, Unmet Challenges, and Translational Perspective

Despite substantial progress in stem cell biology, biomaterials, and regenerative medicine, several important clinical challenges continue to limit the successful translation of stem cell-based therapies into routine medical practice. Although numerous preclinical studies have demonstrated encouraging therapeutic outcomes, reproducibility across clinical studies remains inconsistent. Differences in patient populations, disease stage, genetic background, age, and underlying pathological conditions may contribute to substantial variability in treatment responses [56,102,103,311,312]. Another major challenge involves variability associated with stem cell preparation and manufacturing processes. Cell source, donor characteristics, isolation methods, culture conditions, expansion protocols, and storage procedures may influence cell quality and biological behavior, ultimately affecting therapeutic efficacy [56,80,300,301,302]. Furthermore, achieving large-scale production while maintaining genomic stability, differentiation potential, and reproducibility remains difficult for widespread clinical implementation [283,284,285,286,287,288,289,290,300].

Cell delivery and long-term engraftment also remain important obstacles. Following administration, a substantial proportion of transplanted cells demonstrate limited survival because of inflammatory responses, poor vascularization, immune-mediated effects, and inadequate integration with host tissues [291,292,293,294,295,296,297]. As discussed throughout this review, biomaterial-assisted systems and nano-enabled approaches have emerged as promising strategies to improve cell retention, microenvironmental support, and delivery precision [44,46,47,48,49,50,51,53,54,55,56,82,83,84,86,87,94,313,314].

In addition, regulatory and economic considerations represent significant barriers to broader clinical adoption. Standardization of manufacturing protocols, long-term safety monitoring, regulatory approval pathways, and treatment costs continue to affect the implementation of stem cell-based interventions [315,316,317,318,319]. These challenges become increasingly important as therapies move from experimental studies toward personalized and large-scale clinical applications.

Collectively, these findings suggest that future progress will likely depend not only on advances in stem cell biology itself but also on the successful integration of biomaterials science, nanotechnology, gene-editing approaches, and standardized translational frameworks. Addressing these limitations may improve therapeutic reproducibility, enhance patient outcomes, and accelerate the transition of stem cell therapies into clinically effective regenerative strategies [55,56,57,315,316,317,318,319,320,321,322,323,324,325,326,327,328,329].

## 7. Conclusions

Although stem cell therapies hold transformative potential, their clinical translation remains constrained by multiple biological, technical, and safety challenges. Addressing issues such as uncontrolled differentiation, genetic instability, immune responses, and limited integration is critical for advancing the field. Future progress will depend on improved control of stem cell behavior, development of safer and more efficient delivery systems, and integration of emerging technologies such as gene editing, biomaterials, and cell-free approaches to enhance therapeutic efficacy and safety.

## 8. Future Aspects of Stem Cell Therapy

### 8.1. Regenerative Medicine and Tissue Engineering

Regenerative medicine and tissue engineering integrate stem cell biology with biomaterial science to develop constructs that can restore, maintain, or improve damaged tissues, a paradigm that has evolved significantly over the past decade [57,330]. This multidisciplinary field leverages advances in stem cell isolation, expansion, and differentiation alongside innovations in scaffold design to create functional tissue replacements [331,332]. Despite these advances, replicating the complexity of native extracellular matrices remains a major challenge, particularly in terms of dynamic biochemical signaling and mechanical heterogeneity [333,334].

Effective tissue engineering relies on the interplay between stem cells and engineered scaffolds, where biomaterials provide the necessary physical support and biochemical cues for cell adhesion, proliferation, and differentiation [335,336]. Materials such as hydrogels, ceramics, and synthetic polymers have been optimized to mimic tissue-specific mechanical properties, which is essential for successful integration into host tissues [332,334]. In addition, surface modifications and incorporation of bioactive molecules enhance control over stem cell fate and improve regenerative outcomes [333,336]. Controlled release of growth factors from scaffolds has been shown to significantly enhance differentiation efficiency and tissue maturation [331,335].

A major limitation in engineering complex or thick tissues is the establishment of functional vascular networks capable of supplying oxygen and nutrients. Strategies such as the incorporation of endothelial cells, the delivery of angiogenic factors, and pre-vascularized scaffold designs have demonstrated improved neovascularization and tissue survival in preclinical models [337,338,339]. Co-culture systems combining stem cells with vascular progenitors further enhance the formation of capillary-like structures and improve functional integration [331,336].

Recent advances in 3D bioprinting and microfabrication have significantly improved the precision of tissue construction, enabling the development of complex architectures with spatially controlled cell distributions [57,334]. These technologies allow customization of scaffold geometry to better replicate native tissue structures [335,339]. However, challenges related to scalability, immune compatibility, and long-term functionality continue to limit clinical translation [336,338]. Regulatory considerations and the need for standardized manufacturing protocols further complicate clinical implementation, emphasizing the importance of integrating computational modeling with experimental design to optimize tissue constructs and predict in vivo behavior [335,339].

In ocular surface regeneration, emerging strategies aim to improve the therapeutic potential of stem cell-based approaches. MSC licensing has been shown to enhance immunomodulatory and anti-inflammatory properties through preconditioning with factors such as TNF-α, IL-1β, and TGF-β, thereby improving corneal repair and immune regulation [340,341,342]. The generation of MSCs from iPSCs offers a renewable and standardized cell source, addressing variability associated with traditional MSC populations, although challenges related to cost, genetic stability, and scalability remain [343,344,345]. In addition, the establishment of well-characterized umbilical cord-derived MSC banks may provide a reliable and ethically accessible source of cells with defined histocompatibility for ocular therapies [346,347,348].

iPSC-derived corneal therapies represent a promising alternative for addressing challenges such as donor shortages, immune rejection, and genetic disorders. However, improving cell survival and integration in diseased environments remains a critical hurdle. Advanced preclinical models, including immune-compromised and humanized systems, are being developed to better evaluate therapeutic efficacy and immune compatibility, thereby facilitating clinical translation [349,350,351]. In parallel, stem cell applications in macular degeneration are increasingly transitioning from experimental research toward clinical investigation, highlighting the growing relevance of stem cell-based strategies in ophthalmology [352].

### 8.2. Gene Editing and Personalized Therapies

Gene editing has emerged as a transformative approach in regenerative medicine, enabling precise modification of genomic sequences to correct pathogenic mutations and restore cellular function [353]. The integration of gene-editing technologies with stem cell platforms has enabled the development of personalized therapeutic strategies targeting the underlying causes of genetic diseases [320,321].

CRISPR-Cas9 is one of the most widely adopted gene-editing systems due to its efficiency and adaptability [322]. By introducing targeted double-strand breaks that are repaired through endogenous cellular mechanisms, CRISPR enables precise genome modification [323]. When applied to stem cells, this technology allows correction of genetic defects prior to differentiation, improving the safety and efficacy of cell-based therapies [324,325].

Patient-derived iPSCs have become a cornerstone of personalized medicine, enabling disease modeling and development of autologous therapeutic strategies [62]. When coupled with CRISPR-Cas9, these cells can be genetically corrected ex vivo, a strategy that has shown significant promise in preclinical and early clinical studies for conditions such as sickle cell anemia and Duchenne muscular dystrophy [326,327,354]. Advances in genomic sequencing and bioinformatics further enhance the precision of mutation targeting and correction [326,328].

Despite these advances, challenges remain, including off-target effects, delivery efficiency, cost, and large-scale manufacturing limitations [315,316]. Ethical concerns surrounding genome editing and regulatory complexities also present significant barriers [317,329]. Regulatory agencies are actively revising guidelines to ensure rigorous safety standards and transparent clinical trial protocols, thereby fostering responsible translation of these technologies into clinical practice [317]. Interdisciplinary collaborations among molecular biologists, bioengineers, clinicians, and ethicists will be critical in refining gene-editing strategies and addressing the multifaceted challenges ahead [318,319,320].

### 8.3. Immunomodulation and Anti-Inflammatory Applications

MSCs exhibit potent immunomodulatory and anti-inflammatory properties, making them promising candidates for treating autoimmune and chronic inflammatory diseases [355]. MSCs influence both innate and adaptive immunity by inhibiting the proliferation of activated T cells and natural killer cells while promoting regulatory T cell expansion, thereby maintaining immune homeostasis [64]. Key mediators include TGF-β, IL-10, PGE2, and IDO, which suppress pro-inflammatory pathways and modulate immune cell function [356,357].

Preclinical and early clinical studies have demonstrated the potential of MSC-based therapies in conditions such as rheumatoid arthritis, multiple sclerosis, inflammatory bowel disease, and systemic lupus erythematosus, where they contribute to reduced inflammation and improved clinical outcomes [355,358]. However, variability among MSC sources and lack of standardized protocols remain significant challenges, highlighting the need for large-scale clinical trials and optimized manufacturing processes [64,357].

### 8.4. Neuroregeneration and Treatment of Neurodegenerative Disorders

Stem cell-based neuroregeneration aims to repair and replace damaged neural tissue in conditions such as Parkinson’s disease, Alzheimer’s disease, and spinal cord injuries [147]. Stem cells contribute to neural repair through both differentiation into neuronal and glial cells and secretion of neurotrophic factors that support neuronal survival and synaptic plasticity [359,360]. Key factors such as brain-derived neurotrophic factor (BDNF) and glial cell-derived neurotrophic factor (GDNF) promote neuronal survival and regeneration, while transplanted cells may integrate into neural circuits and support functional recovery [361,362]. In addition, stem cell therapies can modulate the inflammatory microenvironment, reduce secondary damage, and promote tissue repair [363]. Preclinical studies have demonstrated functional improvements in neurodegenerative disease models, including recovery of motor function in Parkinson’s disease and partial restoration of function in spinal cord injury models [364,365,366]. Early phase clinical trials have further supported the safety and potential efficacy of these approaches [367].

Despite these advances, challenges such as limited cell survival, immune responses, and integration into complex neural circuits remain significant [368,369,370]. In addition, the blood–brain barrier presents a major obstacle to efficient delivery. Emerging strategies, including biomaterial scaffolds, preconditioning techniques, and combinatorial therapies integrating gene editing and neuroprotective agents, aim to overcome these limitations [362,363,365].

Advances in RGC replacement and axon regeneration have further expanded the scope of visual neuroregeneration. Differentiation of pluripotent stem cells into RGC-like cells and their integration into retinal circuitry represent promising approaches for treating diseases such as glaucoma and optic neuropathies [202,371,372,373]. Although this area is still in its infancy, it is expected to play an increasingly important clinical role in conditions where RGCs are irreversibly lost, such as Leber’s hereditary optic neuropathy, glaucoma, and neurofibromatosis-related optic pathway gliomas.

Biotechnology and pharmaceutical companies are increasingly devoted to moving stem cell therapies from research into clinical and industrial use. By trying to build on decades of hematopoietic stem cell transplantation, the field now includes mesenchymal, pluripotent, and gene-modified cell products. Current priorities include establishing standardized GMP-compliant systems for cell expansion, differentiation, and cryopreservation; scaling the manufacture of allogeneic MSC products and pluripotent stem cell-derived tissues; and applying technologies such as bioreactors, 3D biomaterials, and nanotechnology to improve product consistency and potency. Large biopharmaceutical companies and specialized regenerative medicine firms are pursuing clinical trials for hematologic malignancies, inherited anemias, myocardial infarction, critical limb ischemia, stroke, spinal cord injury, osteoarthritis, and neurodegenerative diseases, often through partnerships that combine academic innovation with industrial manufacturing and regulatory expertise. Various organizations and dedicated stem cell companies, including MSC developers, iPSC platforms, and cell engineering startups, further support pipeline growth. Together, these efforts are expected to expand the stem cell manufacturing market as more therapies advance into late-stage trials and regulatory pathways for complex cell products become more clearly defined.

In summary, the field of visual regeneration has made significant progress over the past two decades; however, substantial challenges remain before consistent and clinically meaningful functional recovery can be achieved. Collectively, these advances highlight the transformative potential of stem cell-based therapies. Nonetheless, overcoming limitations related to safety, scalability, immune compatibility, and functional integration will be essential for their successful translation into routine clinical practice.

## Figures and Tables

**Table 1:** Major translational limitations associated with pluripotent stem cell-based therapies.

Issue	Embryonic Stem Cells (ESCs)	Induced Pluripotent Stem Cells (iPSCs)	References
Ethical concerns	Ethical concerns related to embryo use remain an important challenge	Lower ethical concerns compared with ESCs	[73]
Genomic instability	May occur during long-term culture	Can occur during cell reprogramming	[72,73]
Tumorigenic risk	Risk of teratoma formation from undifferentiated cells	Risk of teratoma formation and abnormal cell growth	[73]
Differentiation control	Difficulties in obtaining fully controlled differentiation	Variability in differentiation efficiency	[72,73]
Manufacturing challenges	Standardization and scalability concerns	Complex reprogramming and production processes	[72]
Immune-related concerns	Possible immune rejection in allogeneic applications	Lower risk in autologous applications	[72]

**Table 2:** Comparative summary of stem cell delivery strategies and their translational characteristics.

Delivery Strategy	Main Characteristics	Advantages	Limitations	Current Translational Status	References
Direct injection	Cells are administered directly into target tissues through local or systemic routes	Simple procedure, minimally invasive, widely used clinically	Rapid cell washout, low retention, limited control over local microenvironment	Clinically established	[43,87,88]
Biomaterial-assisted systems	Cells are delivered within supportive materials such as hydrogels, cryogels, or scaffold-based matrices	Improved cell survival and retention, structural support, and localized delivery	Material degradation, scaffold optimization challenges, and possible immune responses	Emerging clinical applications	[44,46,47,48,56,80,81,82,83,84]
Nano-enabled systems	Nanomaterials are integrated to improve targeting, tracking, and delivery performance	Controlled release, cell tracking, targeted delivery capabilities	Regulatory challenges, reproducibility concerns, and long-term biosafety issues	Primarily preclinical and early clinical development	[54,55,94,95,96,97,98,99,100,101]

**Table 3:** Bioactive molecules involved in stem cell-derived paracrine effects across different disease systems.

Disease System	Major Bioactive Molecules	Potential Therapeutic Effects	References
Neurological disorders	BDNF, GDNF, NGF, miR-124, miR-133b	Neuroprotection, synaptic repair, neuronal survival	[123,124,125,126,127,128,129]
Cardiovascular diseases	VEGF, IGF-1, HGF, miR-21, miR-126	Angiogenesis, reduced apoptosis, cardiac repair	[213,214,215,216,217,218,219,220,221,222,223,224,225,226]
Orthopedic diseases	TGF-β, BMP-2, IL-10, miR-140	Cartilage repair, bone regeneration, anti-inflammatory effects	[227,228,229,230,231,232,233,234,235,236]
Stroke	VEGF, BDNF, miR-126, miR-17-92 cluster	Angiogenesis, neural repair, functional recovery	[119,120,121,122,123,124,125,126,127,128,129,130,131,132,133]
Alzheimer’s disease	BDNF, NGF, IL-10, miR-146a	Neuroprotection, reduction in inflammation	[104,105,106,134,135,136,137,138,139,140,141,142,143,144,145,146,147,148,149,150]
Oncology	TRAIL, IFN-β, cytokines, exosomal miRNAs	Tumor targeting, immune modulation	[244,245,246,247,248]

**Table 4:** Mechanisms of stem cell therapy across different disease systems.

Mechanism	Neurological	Cardiovascular	Orthopedic	Oncology
Immunomodulation	✔️	✔️	✔️	✔️
Paracrine signaling	✔️	✔️	✔️	✔️
Cell replacement	✔️	Limited	Limited	Limited
Angiogenesis	✔️	✔️	✔️	✔️
Targeted delivery	❌	❌	❌	✔️

**Table 5:** Summary of stem cell applications across major disease systems.

Field	Disease	Stem Cell Type	Mechanism	Most Advanced Clinical Phase	Primary Outcome	Landmark Trial (NCT)	Key Limitations	References
Neurological	Parkinson’s disease	iPSC-derived dopaminergic cells	Cell replacement	Phase I/II	Safety, graft survival, dopamine production	jRCT2090220384	Variability, dyskinesia	[98,99,100,101,107,108,109,110]
Neurological	SCI	MSC, NSC	Immunomodulation, regeneration	Phase I/II	Safety, neurological improvement	NCT01772810	Low survival	[94,95,96,97,98,99,100,101]
Neurological	Stroke	MSC	Paracrine signaling, angiogenesis	Phase II	Safety, functional recovery	NCT02448641	Inconsistent efficacy	[103,104,105,106,107,108,109,110,111,112,113]
Neurological	Alzheimer’s disease	MSC, NSC	Immunomodulation	Phase I	Safety, cognitive outcomes	NCT01297218	Limited efficacy	[134,135,136,137,138,139,140,141,142,143,144,145,146,147,148,149,150]
Ophthalmology	Cornea	MSC, iPSC	Tissue repair	Early clinical	Safety, epithelial repair	—	Integration issues	[149,150,151,152,153,154,155,156]
Ophthalmology	AMD	iPSC-derived RPE	Cell replacement	Phase I/II	Safety, visual outcomes	NCT04339764	Immune response	[206,207,208,209,210,211,212]
Cardiovascular	MI	MSC	Paracrine signaling	Phase II	Safety, improvement in EF	NCT00893360	Low retention	[215,216,217,218]
Orthopedic	OA	MSC	Secretome-mediated effects	Phase II/III	Pain reduction, functional improvement	NCT03382938	Variability	[239,240]
Oncology	CSC targeting	Engineered MSC	Targeted delivery	Preclinical/Early clinical	Safety, targeted therapeutic delivery	—	Safety	[247,248]

## Data Availability

The original contributions presented in the study are included in the article; further inquiries can be directed to the corresponding author.

## Abbreviations

The following abbreviations are used in this manuscript:

SCT	Stem Cell Therapy
ESC	Embryonic Stem Cells
iPSCs	Induced Pluripotent Stem Cells
hESC	Human Embryonic Stem Cells
ISCT	International Society for Cell & Gene Therapy
MSCs	Mesenchymal Stromal Cells
VSELs	very small embryonic-like stem cells
ECM	Extracellular Matrix
PEG	Polyethylene Glycol
MRI	Magnetic Resonance Imaging
PET	Positron Emission Tomography
LNPs	Lipid nanoparticles
PD	Parkinson’s Disease
hfVM	Human Fetal Ventral Mesencephalic
PSC	Pluripotent Stem Cell
GMP	Good Manufacturing Practice
SCI	Spinal Cord Injury
NS/PCs	Neural Stem/Progenitor Cells
OPCs	Oligodendrocyte Progenitor Cells
MAPCs	Multipotent Adult Progenitor Cells
AD	Alzheimer’s Disease
LESCs	Limbal Epithelial Stem Cells
LSCD	Limbal Stem Cell Deficiency
BM-MSCs	Bone Marrow-Derived MSCs
ADSCs	Adipose-Derived Stem Cells
IGF-1	Insulin-Like Growth Factor-1
SKSCs	Skin Keratinocyte Stem Cells
OMECs	Oral Mucosal Epithelial Cells
RGCs	Retinal Ganglion Cells
ILM	Inner Limiting Membrane
AMD	Age-Related Macular Degeneration
dAMD	Dry Form of Age-Related Macular Degeneration
RPE	Retinal Pigment Epithelial
CVDs	Cardiovascular And Cerebrovascular Diseases
MI	Myocardial Infarction
EF	Ejection Fraction
CADUCEUS	Cardiosphere-Derived Autologous Stem Cells to Reverse Ventricular Dysfunction
OA	Osteoarthritis
CRISPR	Clustered Regularly Interspaced Short Palindromic Repeats
HSCT	Hematopoietic Stem Cell Transplantation
PBSCs	Peripheral Blood Stem Cells
G-CSF	Granulocyte Colony-Stimulating Factor
CAR	Chimeric Antigen Receptor
NGF	Nerve Growth Factor
HGF	Hepatocyte Growth Factor
VGF	Vascular Endothelial Growth Factor
CSC	Cancer Stem Cell
NSCs	Neural Stem Cells
HSCs	Hematopoietic Stem Cells
GvHD	Graft-Versus-Host Disease
BDNF	Brain-Derived Neurotrophic Factor
GDNF	Glial Cell-Derived Neurotrophic Factor
